# Supplementary material for: The Impact of Exposure to Iodine and Fluorine in Drinking Water on Thyroid Health and Intelligence in School-Age Children: A Cross-Sectional Investigation
Source: Nutrients. 2024 Aug 31;16(17):2913. doi: 10.3390/nu16172913 (PMC11397114; doi:10.3390/nu16172913)
Supplement: Supplementary file 1 [file nutrients-16-02913-s001.zip › nutrients-3123097-supplementary.pdf]

Table S1. Basic characteristics of study population.

Variable	Level	WI-WF group				*p*	Overall	UI-WF group				*p*	Overall
		N-H	N-L	N-M	H-H			L-L	L-H	H-L	H-H		
Sample size		100	99	100	100		399	115	22	93	135		365
Intelligence degree (%)	Dull normal	2 (2.00)	2 (2.04)	2 (2.00)	3 (3.00)	0.002**	9 (2.26)	2 (1.75)	0 (0.00)	1 (1.08)	6 (4.44)	0.314	9 (2.47)
	Excellent	10 (10.00)	7 (7.14)	7 (7.00)	8 (8.00)		32 (8.04)	12 (10.53)	0 (0.00)	8 (8.60)	10 (7.41)		30 (8.24)
	High normal	16 (16.00)	38 (38.78)	29 (29.00)	19 (19.00)		102 (25.63)	34 (29.82)	8 (36.36)	17 (18.28)	34 (25.19)		93 (25.55)
	Marginal	4 (4.00)	0 (0.00)	1 (1.00)	3 (3.00)		8 (2.01)	2 (1.75)	1 (4.55)	2 (2.15)	2 (1.48)		7 (1.92)
	Normal	39 (39.00)	29 (29.59)	31 (31.00)	54 (54.00)		153 (38.44)	33 (28.95)	9 (40.91)	41 (44.09)	56 (41.48)		139 (38.19)
	Superior	29 (29.00)	22 (22.45)	30 (30.00)	13 (13.00)		94 (23.62)	31 (27.19)	4 (18.18)	24 (25.81)	27 (20.00)		86 (23.63)
Thyroid antibody positive (%)	Yes	2 (2.00)	2 (2.02)	0 (0.00)	2 (2.00)	0.591	6 (1.50)	1 (0.87)	0 (0.00)	2 (2.15)	3 (2.22)	0.776	6 (1.64)
	No	98 (98.00)	97 (97.98)	100 (100.00)	98 (98.00)		393 (98.50)	114 (99.13)	22 (100.00)	91 (97.85)	132 (97.78)		359 (98.36)
Thyroid abnormal (%)	No	94 (94.00)	81 (81.82)	89 (89.00)	51 (51.00)	<.001**	315 (78.95)	99 (86.09)	22 (100.00)	79 (84.95)	88 (65.19)	<0.01**	288 (78.90)
	Yes	6 (6.00)	18 (18.18)	11 (11.00)	49 (49.00)		84 (21.05)	16 (13.91)	0 (0.00)	14 (15.05)	47 (34.81)		77 (21.10)
Thyroid diseases (%)	Hyperthyroidism/ subclinical hyperthyroidism	3 (3.00)	2 (2.20)	6 (6.00)	6 (6.00)	0.098	17 (4.35)	4 (3.54)	0 (0.00)	4 (4.35)	8 (6.15)	0.425	16 (4.48)
	Hypothyroidism/ subclinical	1 (1.00)	5 (5.49)	1 (1.00)	6 (6.00)		13 (3.32)	2 (1.77)	0 (0.00)	7 (7.61)	3 (2.31)		12 (3.36)

	hypothyroidism												
	Thyroid hormone resistance	0 (0.00)	2 (2.20)	1 (1.00)	0 (0.00)		3 (0.77)	1 (0.88)	0 (0.00)	0 (0.00)	1 (0.77)		2 (0.56)
	Normal	96 (96.00)	82 (90.11)	92 (92.00)	88 (88.00)		358 (91.56)	106 (93.81)	22 (100.00)	81 (88.04)	118 (90.77)		327 (91.60)
Drinking water type (%)	Tap water	23 (23.00)	30 (31.25)	26 (26.80)	52 (55.32)	<.001**	131 (33.85)	27 (24.11)	9 (40.91)	21 (23.60)	66 (50.77)	<0.01 **	123 (34.84)
	Hand press well water	0 (0.00)	0 (0.00)	0 (0.00)	2 (2.13)		2 (0.52)	0 (0.00)	0 (0.00)	0 (0.00)	1 (0.77)		1 (0.28)
	Protected well	8 (8.00)	3 (3.13)	4 (4.12)	3 (3.19)		18 (4.65)	5 (4.46)	0 (0.00)	6 (6.74)	4 (3.08)		15 (4.25)
	Unprotected well	2 (2.00)	1 (1.04)	0 (0.00)	0 (0.00)		3 (0.78)	0 (0.00)	0 (0.00)	1 (1.12)	2 (1.54)		3 (0.85)
	Surface water	3 (3.00)	2 (2.08)	2 (2.06)	5 (5.32)		12 (3.10)	1 (0.89)	0 (0.00)	4 (4.49)	6 (4.62)		11 (3.12)
	Bottled water	60 (60.00)	57 (59.38)	61 (62.89)	25 (26.60)		203 (52.45)	74 (66.07)	12 (54.55)	55 (61.80)	44 (33.85)		185 (52.41)
	Others	4 (4.00)	3 (3.13)	4 (4.12)	7 (7.45)		18 (4.65)	5 (4.46)	1 (4.55)	2 (2.25)	7 (5.38)		15 (4.25)
Eating habits (%)	Vegetarian-oriented	9 (9.09)	11 (11.46)	18 (18.56)	19 (19.59)	0.082	57 (14.65)	11 (9.82)	4 (18.18)	13 (14.61)	23 (17.42)	0.379	51 (14.37)
	Give priority to meat	21 (21.21)	14 (14.58)	11 (11.34)	9 (9.28)		55 (14.14)	18 (16.07)	1 (4.55)	15 (16.85)	14 (10.61)		48 (13.52)
	Meat-vegetarian equilibrium	69 (69.70)	71 (73.96)	68 (70.10)	69 (71.13)		277 (71.21)	83 (74.11)	17 (77.27)	61 (68.54)	95 (71.97)		256 (72.11)
Sea foods (%)	Yes	6 (6.00)	20 (20.83)	11 (11.34)	3 (3.09)	<.001**	40 (10.26)	15 (13.39)	1 (4.55)	9 (10.00)	10 (7.58)	0.383	35 (9.83)
	No	94 (94.00)	76 (79.17)	86 (88.66)	94 (96.91)		350 (89.74)	97 (86.61)	21 (95.45)	81 (90.00)	122 (92.42)		321 (90.17)
Salted product (%)	Yes	8 (8.00)	14 (14.58)	10 (10.31)	15 (15.63)	0.314	47 (12.08)	13 (11.61)	3 (13.64)	9 (10.00)	17 (12.88)	0.918	42 (11.80)
	No	92 (92.00)	82 (85.42)	87 (89.69)	81 (84.38)		342 (87.92)	99 (88.39)	19 (86.36)	81 (90.00)	115 (87.12)		314 (88.20)
Kelp	Yes	5 (5.00)	8 (8.33)	10 (10.31)	11 (11.46)	0.397	34 (8.74)	4 (3.57)	1 (4.55)	8 (8.89)	15 (11.36)	0.136	28 (7.87)

seaweed soup (%)	No	95 (95.00)	88 (91.67)	87 (89.69)	85 (88.54)		355 (91.26)	108 (96.43)	21 (95.45)	82 (91.11)	117 (88.64)		328 (92.13)
Drinking taste (%)	Salty	16 (16.00)	13 (13.54)	13 (13.40)	10 (10.42)		52 (13.37)	15 (13.39)	4 (18.18)	14 (15.56)	12 (9.09)		45 (12.64)
	Moderately salted	54 (54.00)	62 (64.58)	60 (61.86)	48 (50.00)	0.149	224 (57.58)	66 (58.93)	12 (54.55)	54 (60.00)	75 (56.82)	0.602	207 (58.15)
	Mild	30 (30.00)	21 (21.88)	24 (24.74)	38 (39.58)		113 (29.05)	31 (27.68)	6 (27.27)	22 (24.44)	45 (34.09)		104 (29.21)
Salt type (%)	Non-iodized salt	15 (15.00)	12 (12.50)	16 (16.49)	19 (20.00)		62 (15.98)	24 (21.43)	5 (22.73)	6 (6.67)	21 (16.03)		56 (15.77)
	Iodized salt	65 (65.00)	64 (66.67)	58 (59.79)	43 (45.26)	0.068	230 (59.28)	65 (58.04)	12 (54.55)	61 (67.78)	74 (56.49)	0.112	212 (59.72)
	Both	20 (20.00)	20 (20.83)	23 (23.71)	33 (34.74)		96 (24.74)	23 (20.54)	5 (22.73)	23 (25.56)	36 (27.48)		87 (24.51)
Tea drinking (%)	Yes	3 (3.00)	4 (4.17)	4 (4.12)	8 (8.33)	0.381	19 (4.88)	2 (1.79)	1 (4.55)	6 (6.67)	8 (6.06)	0.260	17 (4.78)
	No	97 (97.00)	92 (95.83)	93 (95.88)	88 (91.67)		370 (95.12)	110 (98.21)	21 (95.45)	84 (93.33)	124 (93.94)		339 (95.22)
Weekly passive smoking frequency (%)	0 days	37 (37.00)	48 (50.00)	40 (41.24)	41 (42.71)		166 (42.67)	47 (41.96)	9 (40.91)	41 (45.56)	54 (40.91)		151 (42.42)
	1-2 days	12 (12.00)	16 (16.67)	25 (25.77)	14 (14.58)		67 (17.22)	16 (14.29)	4 (18.18)	18 (20.00)	20 (15.15)		58 (16.29)
	3-5 days	11 (11.00)	7 (7.29)	4 (4.12)	2 (2.08)	0.022*	24 (6.17)	9 (8.04)	3 (13.64)	6 (6.67)	4 (3.03)	0.111	22 (6.18)
	Almost every day	32 (32.00)	15 (15.63)	18 (18.56)	28 (29.17)		93 (23.91)	30 (26.79)	4 (18.18)	23 (25.56)	32 (24.24)		89 (25.00)
	Unclear	8 (8.00)	10 (10.42)	10 (10.31)	11 (11.46)		39 (10.03)	10 (8.93)	2 (9.09)	2 (2.22)	22 (16.67)		36 (10.11)
Weekly physical exercise (%)	More than 6 times	10 (10.10)	18 (18.95)	25 (25.77)	26 (27.08)		79 (20.41)	28 (25.45)	4 (18.18)	16 (17.78)	29 (21.97)		77 (21.75)
	3-5 times	46 (46.46)	49 (51.58)	46 (47.42)	42 (43.75)	0.029*	183 (47.29)	51 (46.36)	8 (36.36)	45 (50.00)	59 (44.70)	0.719	163 (46.05)
	One or two times	28 (28.28)	22 (23.16)	22 (22.68)	21 (21.88)		93 (24.03)	21 (19.09)	6 (27.27)	22 (24.44)	34 (25.76)		83 (23.45)
	A fat lot	15 (15.15)	6 (6.32)	4 (4.12)	7 (7.29)		32 (8.27)	10 (9.09)	4 (18.18)	7 (7.78)	10 (7.58)		31 (8.76)
Stress (%)	Less	37 (37.37)	58 (60.42)	58 (59.79)	62 (64.58)	<.001**	215 (55.41)	58 (52.25)	10 (45.45)	53 (58.89)	78 (59.09)	0.399	199 (56.06)

	Common	49 (49.49)	37 (38.54)	38 (39.18)	30 (31.25)		154 (39.69)	46 (41.44)	12 (54.55)	31 (34.44)	50 (37.88)		139 (39.15)
	More	13 (13.13)	1 (1.04)	1 (1.03)	4 (4.17)		19 (4.90)	7 (6.31)	0 (0.00)	6 (6.67)	4 (3.03)		17 (4.79)
Anxious (%)	Less	69 (69.70)	76 (79.17)	70 (72.16)	63 (65.63)	0.581	278 (71.65)	84 (75.68)	16 (72.73)	67 (74.44)	89 (67.42)	0.197	256 (72.11)
	Mild	24 (24.24)	15 (15.63)	21 (21.65)	23 (23.96)		83 (21.39)	21 (18.92)	5 (22.73)	13 (14.44)	36 (27.27)		75 (21.13)
	Moderate	5 (5.05)	5 (5.21)	4 (4.12)	8 (8.33)		22 (5.67)	4 (3.60)	1 (4.55)	7 (7.78)	7 (5.30)		19 (5.35)
	Serious	1 (1.01)	0 (0.00)	2 (2.06)	2 (2.08)		5 (1.29)	2 (1.80)	0 (0.00)	3 (3.33)	0 (0.00)		5 (1.41)
Angry (%)	Never	7 (7.07)	10 (10.42)	9 (9.28)	14 (14.58)	0.492	40 (10.31)	11 (9.91)	0 (0.00)	10 (11.11)	18 (13.64)	0.254	39 (10.99)
	Occasionally	67 (67.68)	68 (70.83)	73 (75.26)	59 (61.46)		267 (68.81)	74 (66.67)	20 (90.91)	63 (70.00)	85 (64.39)		242 (68.17)
	Sometimes	24 (24.24)	17 (17.71)	15 (15.46)	22 (22.92)		78 (20.10)	25 (22.52)	2 (9.09)	15 (16.67)	29 (21.97)		71 (20.00)
	Often	1 (1.01)	1 (1.04)	0 (0.00)	1 (1.04)		3 (0.77)	1 (0.90)	0 (0.00)	2 (2.22)	0 (0.00)		3 (0.85)
Psychic trauma (%)	Yes	11 (11.22)	5 (5.26)	8 (8.25)	8 (8.33)	0.521	32 (8.29)	9 (8.26)	1 (4.55)	8 (8.89)	13 (9.85)	0.869	31 (8.78)
	No	87 (88.78)	90 (94.74)	89 (91.75)	88 (91.67)		354 (91.71)	100 (91.74)	21 (95.45)	82 (91.11)	119 (90.15)		322 (91.22)
Often catch a cold (%)	Yes	28 (28.28)	19 (20.00)	13 (13.40)	14 (14.58)	0.033*	74 (19.12)	21 (19.09)	6 (27.27)	16 (17.78)	24 (18.18)	0.771	67 (18.93)
	No	71 (71.72)	76 (80.00)	84 (86.60)	82 (85.42)		313 (80.88)	89 (80.91)	16 (72.73)	74 (82.22)	108 (81.82)		287 (81.07)
Sick history (%)	Chronic kidney disease	1 (1.01)	0 (0.00)	0 (0.00)	1 (1.04)	0.577	2 (0.52)	0 (0.00)	0 (0.00)	0 (0.00)	1 (0.76)	0.330	1 (0.28)
	Other endocrine diseases	0 (0.00)	1 (1.04)	0 (0.00)	0 (0.00)		1 (0.26)	0 (0.00)	0 (0.00)	1 (1.11)	0 (0.00)		1 (0.28)
	Other autoimmune diseases	2 (2.02)	0 (0.00)	3 (3.09)	2 (2.08)		7 (1.80)	0 (0.00)	0 (0.00)	1 (1.11)	4 (3.03)		5 (1.41)

Head and neck treatment (%)	None	96 (96.97)	95 (98.96)	94 (96.91)	93 (96.88)	0.002**	378 (97.42)	111 (100.00)	22 (100.00)	88 (97.78)	127 (96.21)	0.017*	348 (98.03)
	Radioactive iodine 131	1 (1.01)	0 (0.00)	0 (0.00)	4 (4.17)		5 (1.30)	0 (0.00)	0 (0.00)	0 (0.00)	5 (3.79)		5 (1.42)
	CT examination	2 (2.02)	15 (15.63)	7 (7.37)	9 (9.38)		33 (8.55)	15 (13.76)	0 (0.00)	3 (3.33)	11 (8.33)		29 (8.22)
	Nuclear magnetic resonance examination	2 (2.02)	1 (1.04)	0 (0.00)	0 (0.00)		3 (0.78)	2 (1.83)	0 (0.00)	0 (0.00)	1 (0.76)		3 (0.85)
	None	94 (94.95)	80 (83.33)	88 (92.63)	83 (86.46)		345 (89.38)	92 (84.40)	22 (100.00)	87 (96.67)	115 (87.12)		316 (89.52)
Paternal education level (%)	< high school	73 (73.74)	80 (83.33)	68 (70.10)	80 (84.21)	0.042*	301 (77.78)	90 (81.08)	14 (63.64)	66 (74.16)	109 (82.58)	0.130	279 (78.81)
	≥high school	26 (26.26)	16 (16.67)	29 (29.90)	15 (15.79)		86 (22.22)	21 (18.92)	8 (36.36)	23 (25.84)	23 (17.42)		75 (21.19)
Maternal education level (%)	< high school	79 (79.80)	82 (85.42)	78 (80.41)	84 (88.42)	0.311	323 (83.46)	93 (83.78)	16 (72.73)	74 (83.15)	115 (87.12)	0.375	298 (84.18)
	≥high school	20 (20.20)	14 (14.58)	19 (19.59)	11 (11.58)		64 (16.54)	18 (16.22)	6 (27.27)	15 (16.85)	17 (12.88)		56 (15.82)
Household income (%)	1-50,000 RMB/year	59 (60.20)	47 (50.00)	51 (52.58)	46 (48.94)	0.034*	203 (53.00)	56 (51.38)	15 (68.18)	48 (53.33)	62 (48.06)	0.083	181 (51.71)
	10,000 RMB/year and below	24 (24.49)	22 (23.40)	25 (25.77)	37 (39.36)		108 (28.20)	25 (22.94)	5 (22.73)	30 (33.33)	45 (34.88)		105 (30.00)
	100,000 RMB/year and above	2 (2.04)	1 (1.06)	2 (2.06)	3 (3.19)		8 (2.09)	2 (1.83)	1 (4.55)	3 (3.33)	2 (1.55)		8 (2.29)
	50-100,000 RMB/year	13 (13.27)	24 (25.53)	19 (19.59)	8 (8.51)		64 (16.71)	26 (23.85)	1 (4.55)	9 (10.00)	20 (15.50)		56 (16.00)
Pregnancy conditions	Smoke	4 (4.08)	1 (1.05)	1 (1.03)	0 (0.00)	0.093	6 (1.56)	2 (1.82)	0 (0.00)	2 (2.25)	1 (0.76)	0.277	5 (1.42)
	Drinking	2 (2.04)	1 (1.05)	0 (0.00)	4 (4.21)		7 (1.82)	1 (0.91)	1 (4.55)	0 (0.00)	5 (3.82)		7 (1.99)

(%)	Both none	92 (93.88)	93 (97.89)	96 (98.97)	91 (95.79)		372 (96.62)	107 (97.27)	21 (95.45)	87 (97.75)	125 (95.42)		340 (96.59)
Passive smoking during pregnancy (%)	>6 days/week	16 (16.84)	7 (7.69)	12 (12.37)	20 (21.05)		55 (14.55)	19 (17.12)	1 (4.55)	13 (14.94)	20 (15.75)		53 (15.27)
	3-5days/week	17 (17.89)	13 (14.29)	11 (11.34)	6 (6.32)		47 (12.43)	15 (13.51)	6 (27.27)	13 (14.94)	10 (7.87)		44 (12.68)
	1-2days/week	9 (9.47)	4 (4.40)	11 (11.34)	10 (10.53)	0.019*	34 (8.99)	12 (10.81)	0 (0.00)	5 (5.75)	12 (9.45)	0.353	29 (8.36)
	<1day/week	5 (5.26)	12 (13.19)	5 (5.15)	3 (3.16)		25 (6.61)	5 (4.50)	1 (4.55)	6 (6.90)	9 (7.09)		21 (6.05)
	No passive smoking	48 (50.53)	55 (60.44)	58 (59.79)	56 (58.95)		217 (57.41)	60 (54.05)	14 (63.64)	50 (57.47)	76 (59.84)		200 (57.64)
Delivery conditions (%)	Hypoxia	3 (3.00)	3 (3.03)	4 (4.00)	2 (2.00)		12 (3.01)	4 (3.48)	0 (0.00)	1 (1.08)	5 (3.70)		14 (3.84)
	Dystocia	2 (2.00)	5 (5.05)	6 (6.00)	1 (1.00)		14 (3.51)	4 (3.48)	3 (13.64)	4 (4.30)	3 (2.22)		10 (2.74)
	Premature birth	5 (5.00)	3 (3.03)	3 (3.00)	4 (4.00)	0.128	15 (3.76)	6 (5.22)	0 (0.00)	3 (3.23)	4 (2.96)	0.705	13 (3.56)
	Post term birth	7 (7.00)	2 (2.02)	1 (1.00)	4 (4.00)		14 (3.51)	4 (3.48)	1 (4.55)	3 (3.23)	4 (2.96)		12 (3.29)
	Low birth weight	5 (5.00)	0 (0.00)	0 (0.00)	2 (2.00)		7 (1.75)	4 (3.48)	0 (0.00)	1 (1.08)	2 (1.48)		7 (1.92)
	All none	78 (78.00)	86 (86.87)	86 (86.00)	87 (87.00)		337 (84.46)	93 (80.87)	18 (81.82)	81 (87.10)	117 (86.67)		309 (84.66)

* $p<0.05$; ** $p<0.01$;

a. Statistical value: χ^2 value.

Table s2. Multiple comparisons of differential factors in water Iodine and water fluoride groups.

Comparison items/*p* value	Age	Tvol	FT3	FT4	IQ	UI	UF	5-HT	DA
N-H/N-M	0.12	<0.01**	0.91	0.03*	0.28	0.32	0.06	0.73	0.15
N-H/N-L	0.74	<0.01**	0.13	<0.01**	0.51	0.01*	0.53	0.10	<0.01**
N-H/H-H	0.02*	<0.01**	<0.01**	<0.01**	0.03*	<0.01**	<0.01**	0.17	<0.01**
N-M/N-L	0.06	<0.01**	0.11	0.06	0.68	0.13	0.20	0.17	0.09
N-M/H-H	0.47	<0.01**	<0.01**	0.47	<0.01**	<0.01**	<0.01**	0.08	0.06
N-L/H-H	0.01**	<0.01**	<0.01**	0.30	<0.01**	<0.01**	<0.01**	<0.01**	0.84

*Bonferroni adjusted *p* value<0.05; **Bonferroni adjusted *p* value<0.01.

Table s3. Multiple comparisons of differential factors in the urinary iodine and urinary fluoride groups.

Comparison items/*p* value	Tvol	FT3	FT4	IQ	UI	UF
L-H/L-L	0.19	0.47	0.17	0.03*	0.40	<0.01**
L-H/H-L	0.06	0.46	0.04*	0.22	<0.01**	<0.01**
L-H/H-H	<0.01**	<0.01**	0.58	0.39	<0.01**	0.19
L-L/H-L	0.05	0.96	0.26	0.13	<0.01**	0.08
L-L/H-H	<0.01**	<0.01**	0.15	0.02*	<0.01**	<0.01**
H-L/H-H	<0.01**	<0.01**	0.01*	0.49	<0.01**	<0.01**

*Bonferroni adjusted *p* value<0.05; **Bonferroni adjusted *p* value<0.01.

Table s4. Summary of regression coefficients for the path analysis model between thyroid hormones, monoamine neurotransmitters, and IQ in school-aged children under the combined influence of iodine and fluoride.

X	→	Y	Non-standardized path coefficients	*SE*	*Z* (CR value)	*p*	Standardized path coefficients
FT4	→	DA	0.451	0.232	1.947	0.050^{*}	0.146
FT3	→	DA	1.497	0.661	2.265	0.024^{*}	0.169
TSH	→	NE	20.924	18.586	1.126	0.260	0.081
FT4	→	NE	71.319	16.031	4.449	0.000^{**}	0.321
FT4	→	5-HT	12.235	3.308	3.699	0.000^{**}	0.270
FT3	→	5-HT	16.166	9.433	1.714	0.087	0.125
E	→	IQ	0.003	0.003	0.941	0.346	0.068
DA	→	IQ	-0.049	0.194	-0.251	0.802	-0.023
NE	→	IQ	-0.002	0.002	-0.863	0.388	-0.066
5-HT	→	IQ	0.006	0.014	0.451	0.652	0.043
TSH	→	IQ	-1.136	0.554	-2.049	0.040^{*}	-0.150
FT4	→	IQ	1.227	0.531	2.312	0.021^{*}	0.188
FT3	→	IQ	-2.836	1.396	-2.031	0.042^{*}	-0.152
UF	→	TSH	0.048	0.165	0.293	0.770	0.027
UI	→	TSH	0.000	0.001	0.523	0.601	0.048
UF	→	FT4	0.122	0.191	0.640	0.522	0.059

UI	→	FT4	-0.000	0.001	-0.308	0.758	-0.029
UF	→	FT3	0.002	0.064	0.037	0.970	0.003
UI	→	FT3	-0.001	0.000	-3.404	0.001**	-0.301

Note: → indicates path influence relationships. *p<0.05; **p<0.01.

Table s5. Summary of regression coefficients for the path analysis model of factors influencing thyroid health in schoolchildren under the combined influence of iodine and fluoride.

X	→	Y	Non-standardized path coefficients	*SE*	*Z* (CR value)	*p*	Standardized path coefficients
UF	→	Thyroid nodule	0.021	0.023	0.920	0.358	0.064
UI	→	Thyroid nodule	0.000	0.000	1.545	0.122	0.110
TSH	→	Thyroid nodule	-0.011	0.011	-1.093	0.275	-0.061
FT4	→	Thyroid nodule	0.010	0.009	1.115	0.265	0.063
FT3	→	Thyroid nodule	-0.040	0.025	-1.579	0.114	-0.092
UF	→	TSH	0.111	0.123	0.905	0.365	0.064
UI	→	TSH	0.000	0.001	0.407	0.684	0.029
UF	→	FT4	0.172	0.148	1.163	0.245	0.082
UI	→	FT4	-0.000	0.001	-0.638	0.524	-0.045
UF	→	FT3	-0.024	0.051	-0.458	0.647	-0.031
UI	→	FT3	-0.001	0.000	-4.188	0.000**	-0.283
UF	→	Tvol	0.122	0.092	1.323	0.186	0.078

UI	→	Tvol	0.004	0.000	7.980	0.000**	0.479
TSH	→	Tvol	0.073	0.043	1.707	0.088	0.081
FT4	→	Tvol	0.075	0.036	2.088	0.037*	0.099
FT3	→	Tvol	-0.129	0.102	-1.265	0.206	-0.062

Note: → indicates path influence relationships. *p<0.05; **p<0.01.

Figure S1. Normality test of quantitative variables.

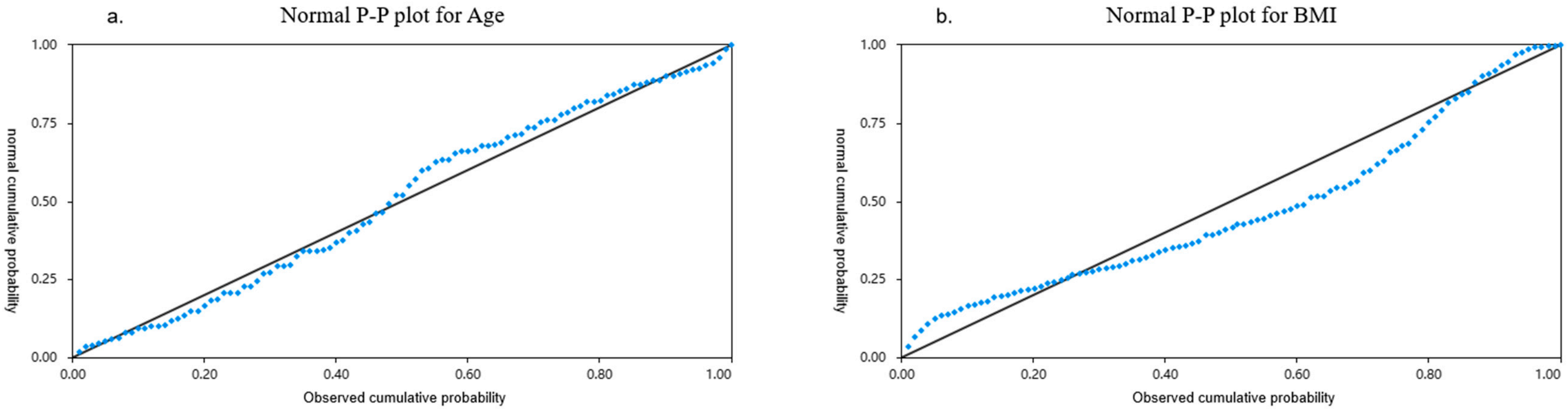

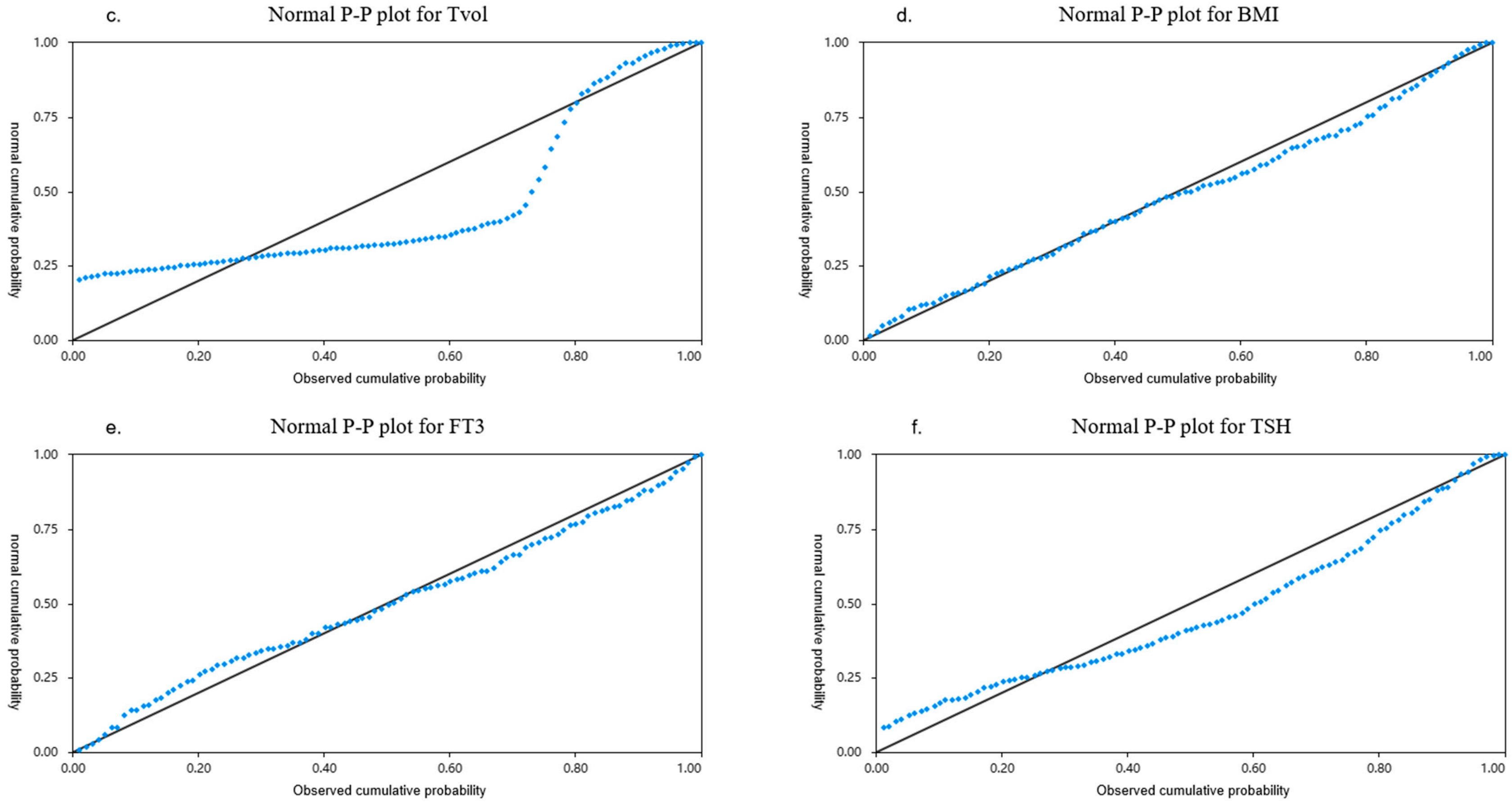
c.
Normal P-P plot for Tvol
normal cumulative probability
Observed cumulative probability
d.
Normal P-P plot for BMI
normal cumulative probability
Observed cumulative probability
e.
Normal P-P plot for FT3
normal cumulative probability
Observed cumulative probability
f.
Normal P-P plot for TSH
normal cumulative probability
Observed cumulative probability

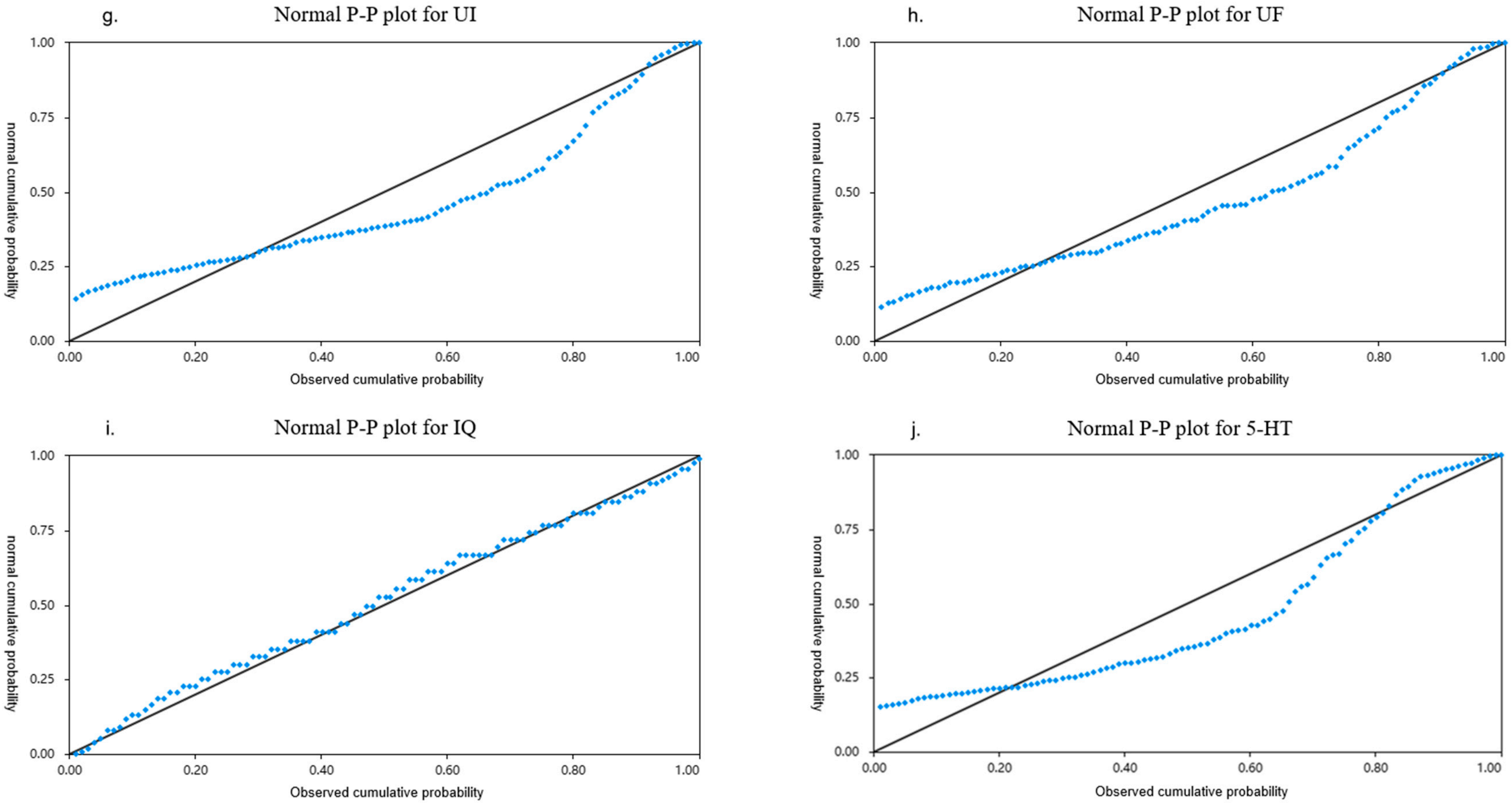
g.
Normal P-P plot for UI
h.
Normal P-P plot for UF
i.
Normal P-P plot for IQ
j.
Normal P-P plot for 5-HT
normal cumulative probability
Observed cumulative probability
0.00
0.25
0.50
0.75
1.00
0.20
0.40
0.60
0.80

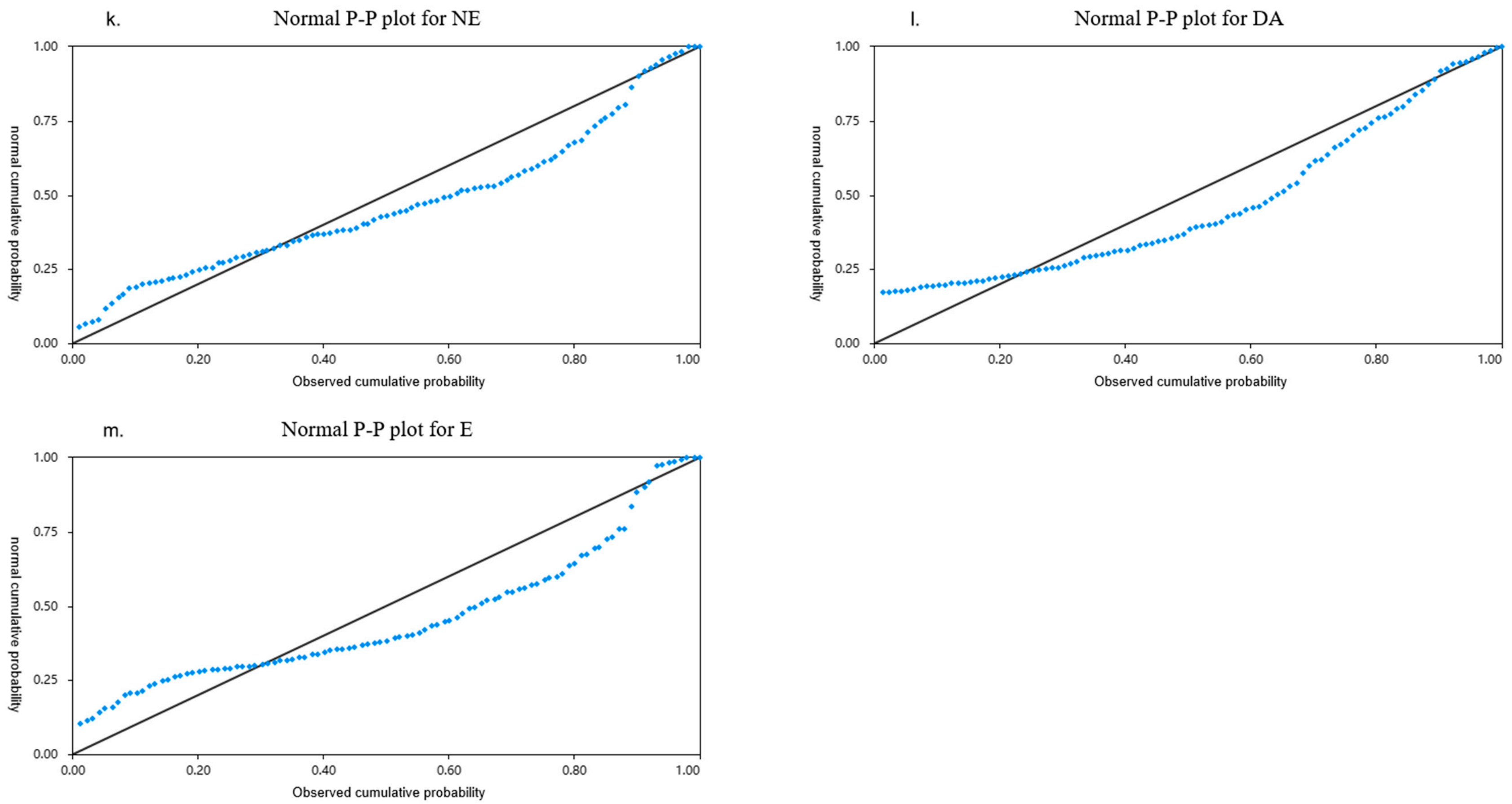

a. Normal P-P plot for Age; b. Normal P-P plot for BMI; c. Normal P-P plot for Tvol; d. Normal P-P plot for FT4; e. Normal P-P plot for FT3; f. Normal P-P plot for TSH; g. Normal P-P plot for UI; h. Normal P-P plot for UF; i. Normal P-P plot for IQ; j. Normal P-P plot for 5-HT; k. Normal P-P plot for NE; l. Normal P-P plot for DA; m. Normal P-P plot for E.

Figure s2. Crude model of urinary iodine RCS curve.

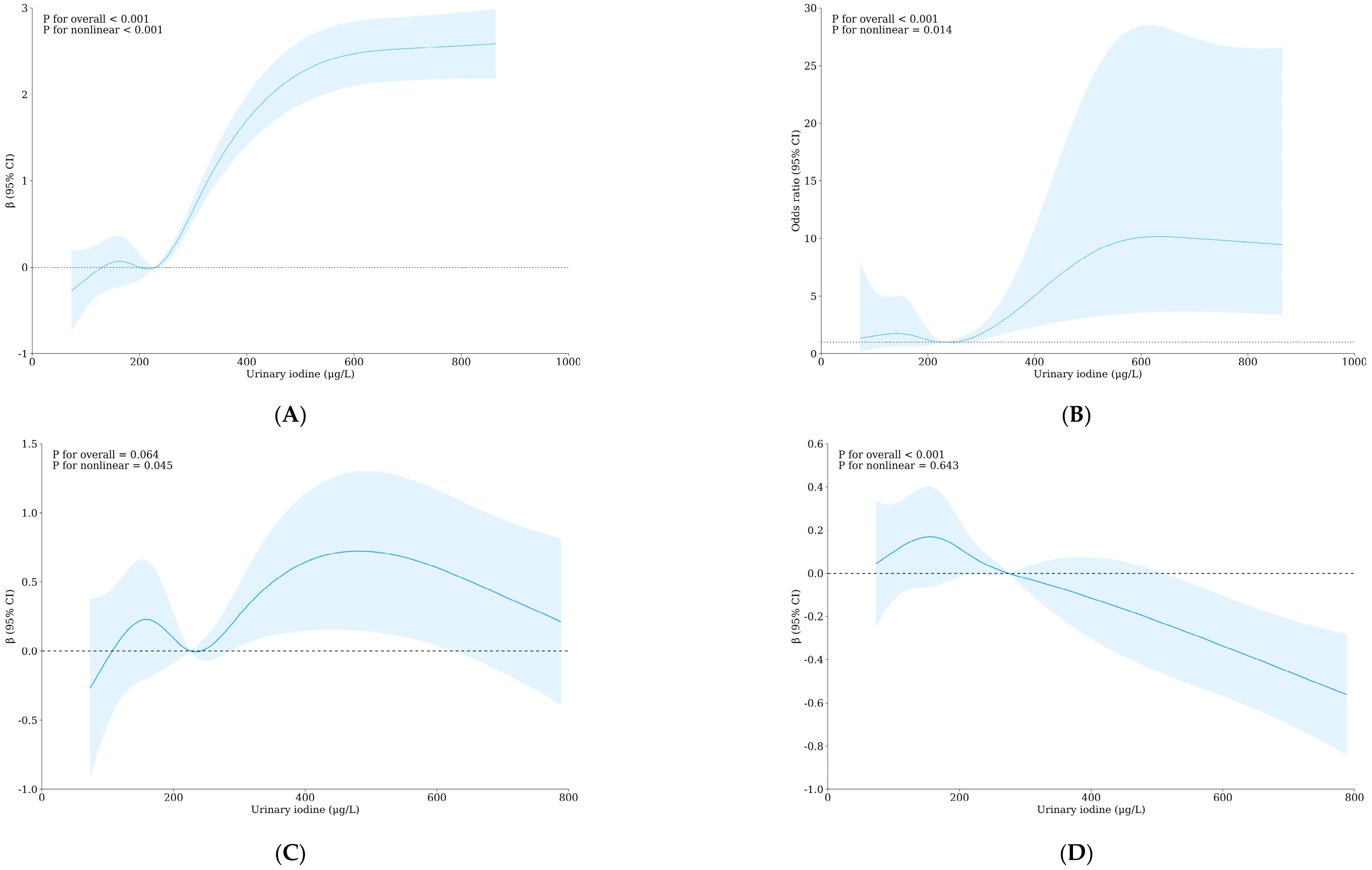

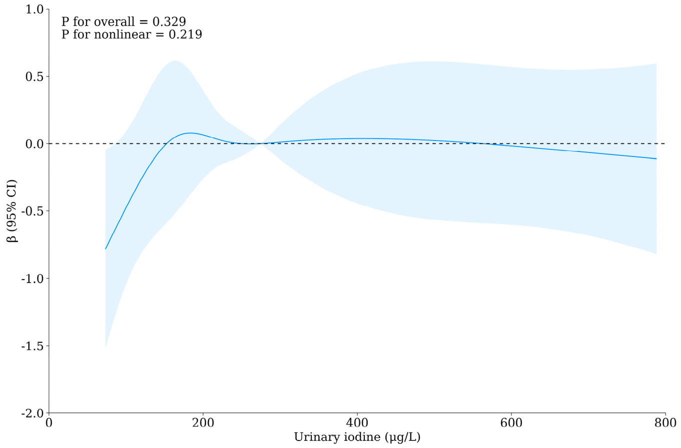
P for overall = 0.329
P for nonlinear = 0.219
β (95% CI)
1.0
0.5
0.0
-0.5
-1.0
-1.5
-2.0
0
200
400
600
800
Urinary iodine (μg/L)

(E)

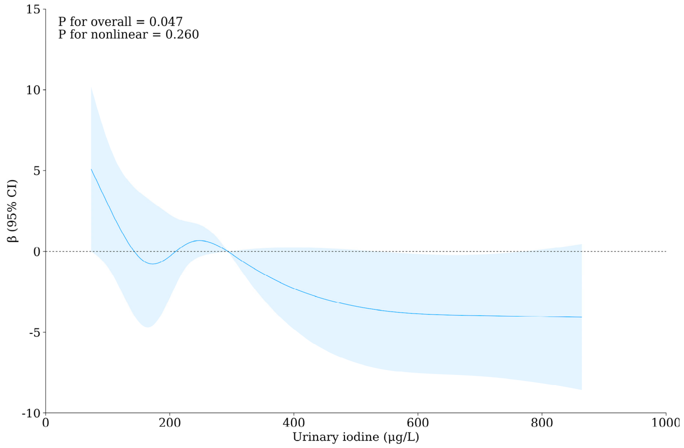
P for overall = 0.047
P for nonlinear = 0.260
β (95% CI)
15
10
5
0
-5
-10
0
200
400
600
800
1000
Urinary iodine (μg/L)

(F)

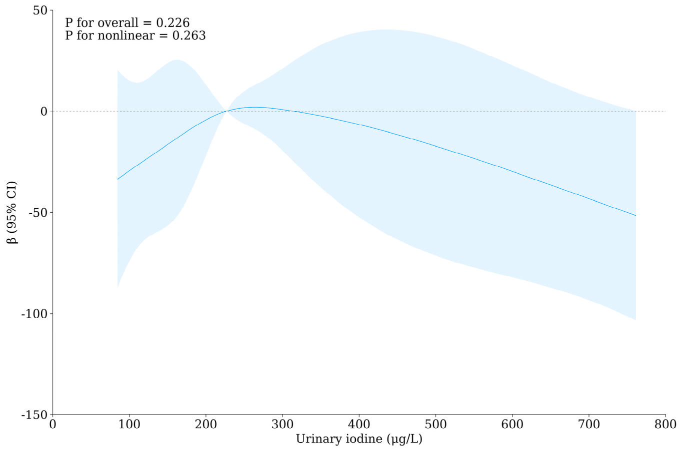
P for overall = 0.226
P for nonlinear = 0.263
β (95% CI)
50
0
-50
-100
-150
0
100
200
300
400
500
600
700
800
Urinary iodine (μg/L)

(G)

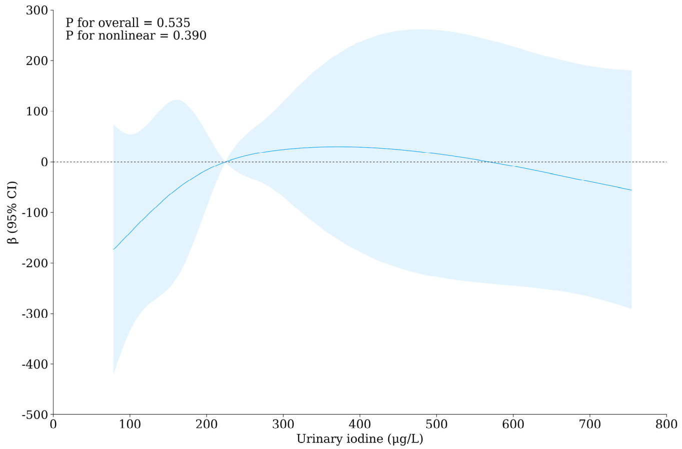
P for overall = 0.535
P for nonlinear = 0.390
β (95% CI)
300
200
100
0
-100
-200
-300
-400
-500
0
100
200
300
400
500
600
700
800
Urinary iodine (μg/L)

(H)

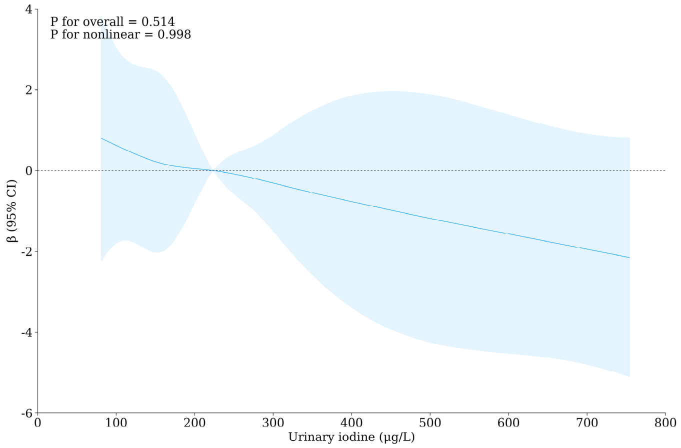

(I)

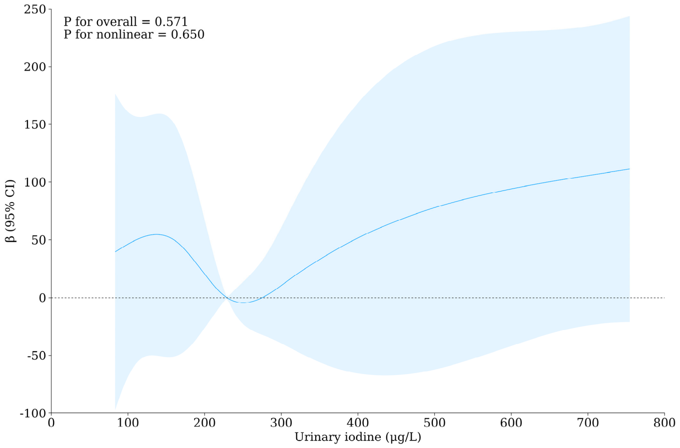

(J)

A. Tvol; B. Thyroid nodule; C. TSH; D. FT3; E. FT4; F. IQ; G. 5-HT; H. NE; I. DA; J. E.

Figure s3. Urinary iodine RCS curve adjustment model.

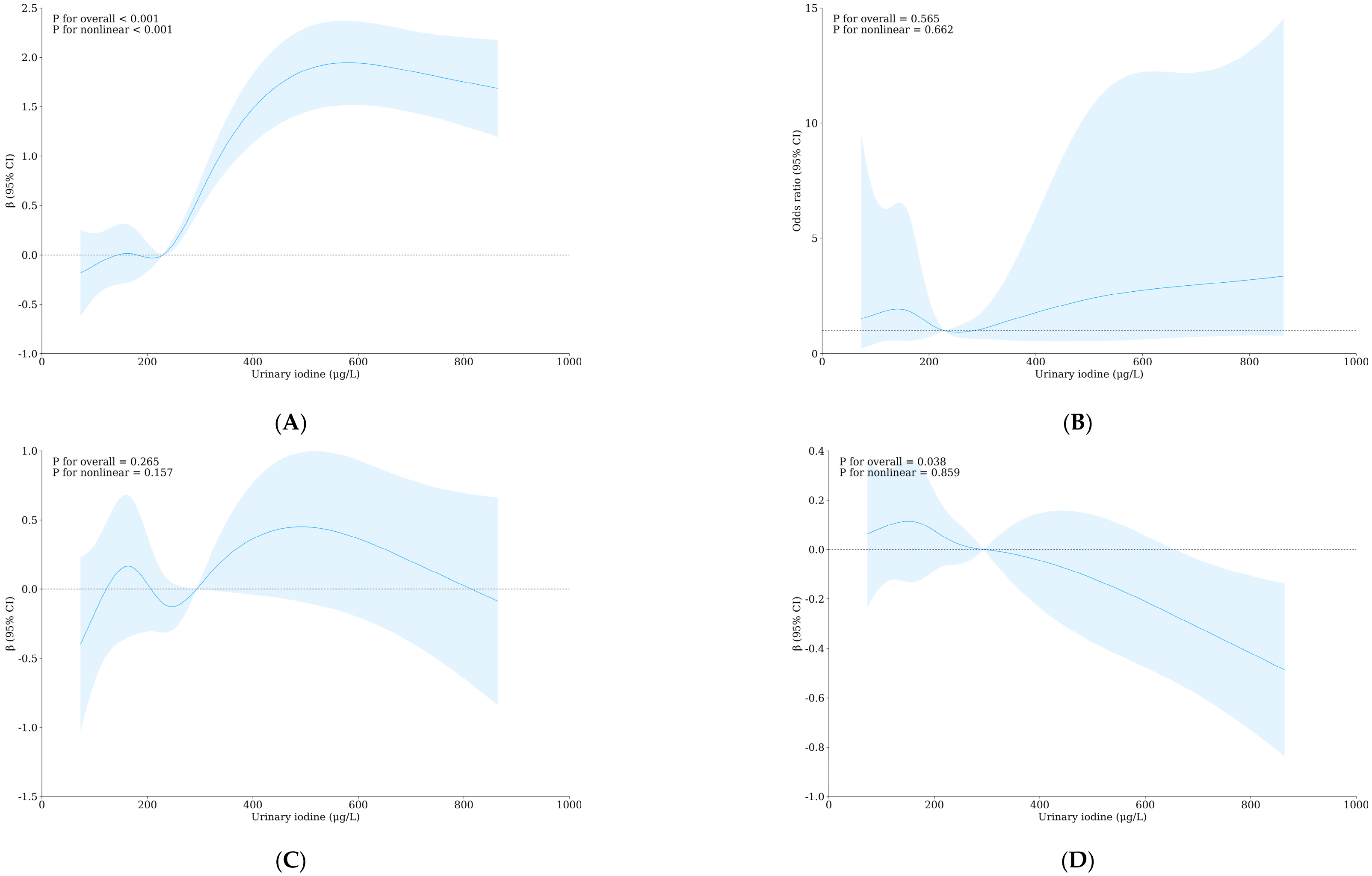

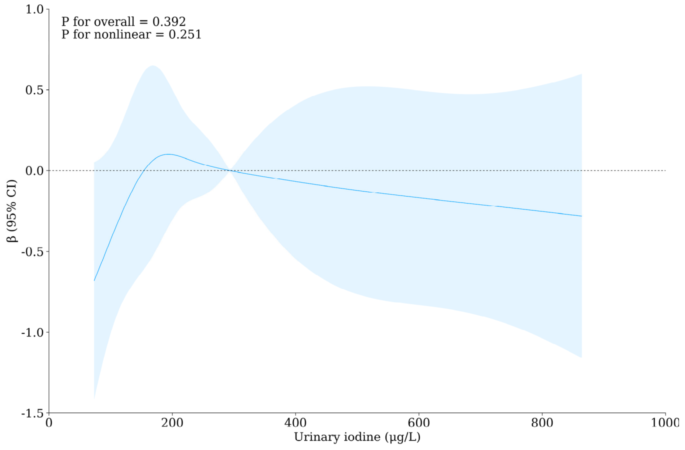
P for overall = 0.392
P for nonlinear = 0.251
β (95% CI)
Urinary iodine (μg/L)

(E)

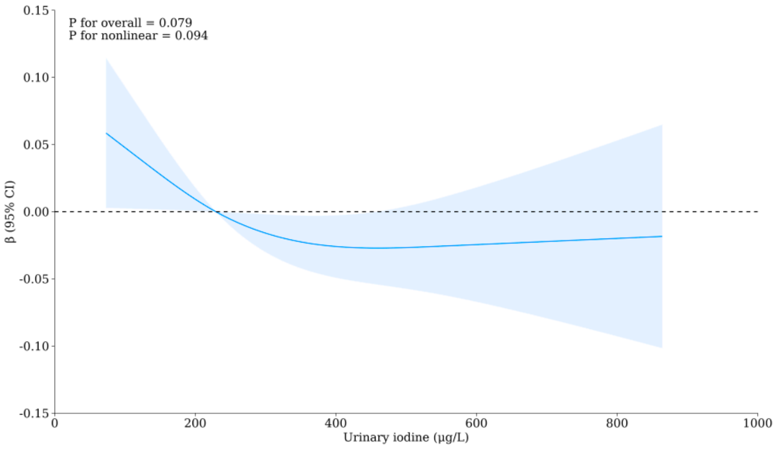
P for overall = 0.079
P for nonlinear = 0.094
β (95% CI)
Urinary iodine (μg/L)

(F)

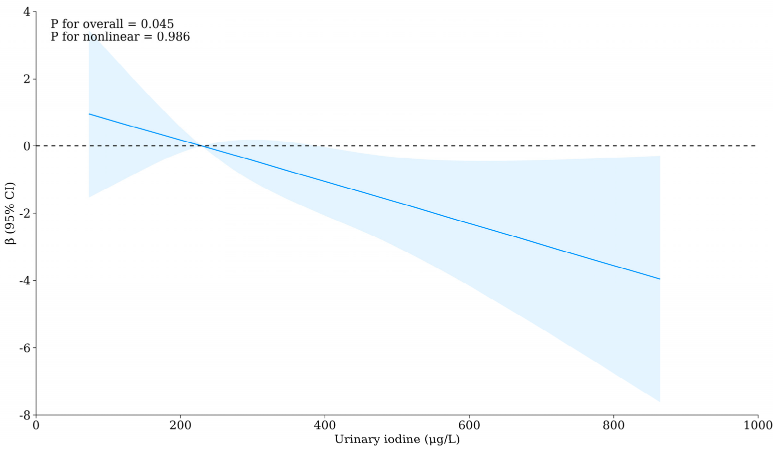
P for overall = 0.045
P for nonlinear = 0.986
β (95% CI)
Urinary iodine (μg/L)

(G)

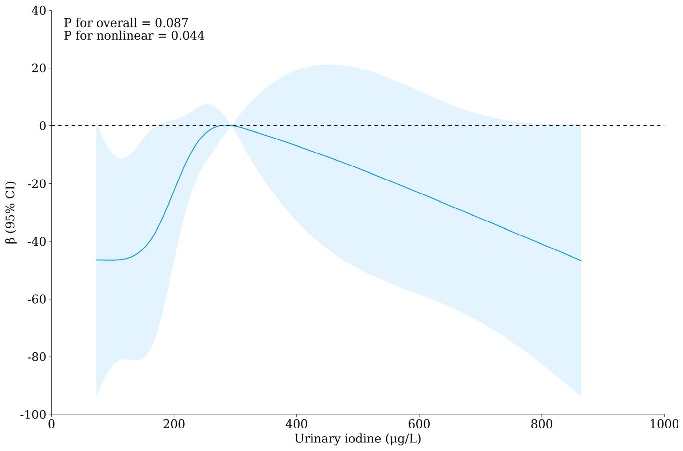
P for overall = 0.087
P for nonlinear = 0.044
β (95% CI)
Urinary iodine (μg/L)

(H)

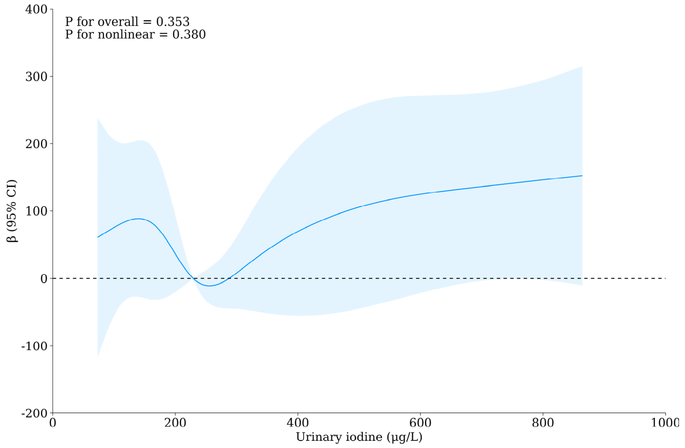

(I)

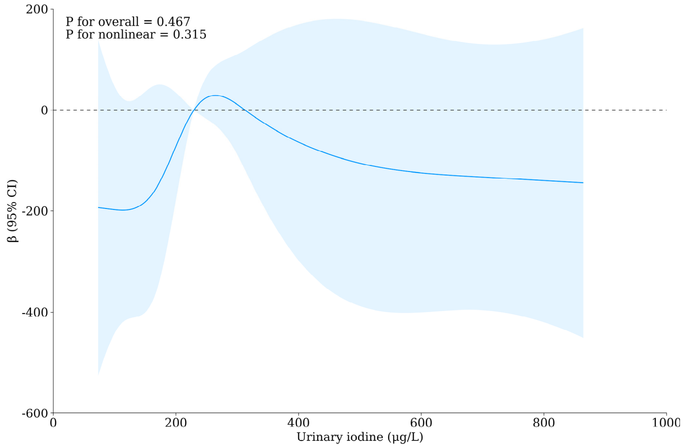

(J)

A. Tvol; B. Thyroid nodule; C. TSH; D. FT3; E. FT4; F. IQ; G. DA; H. 5-HT; I. E; J. NE.

Figure s4. Crude model of urinary fluoride RCS curve.

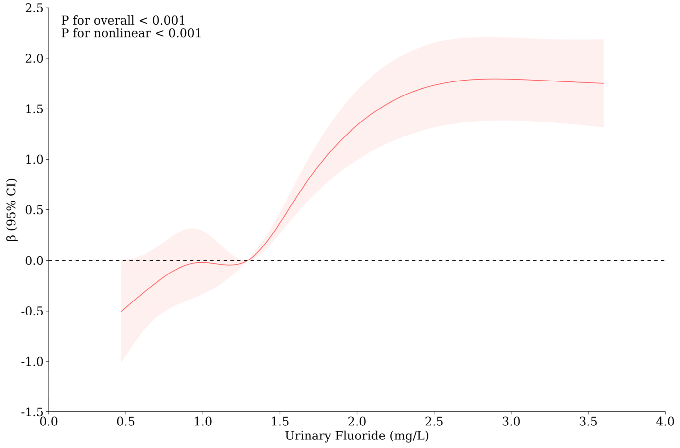

(**A**)

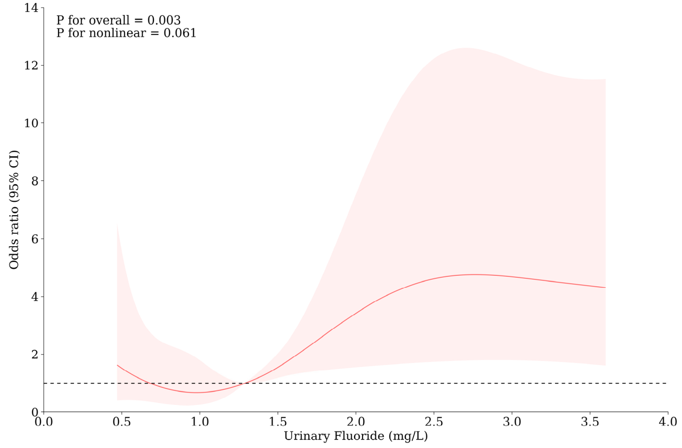

(**B**)

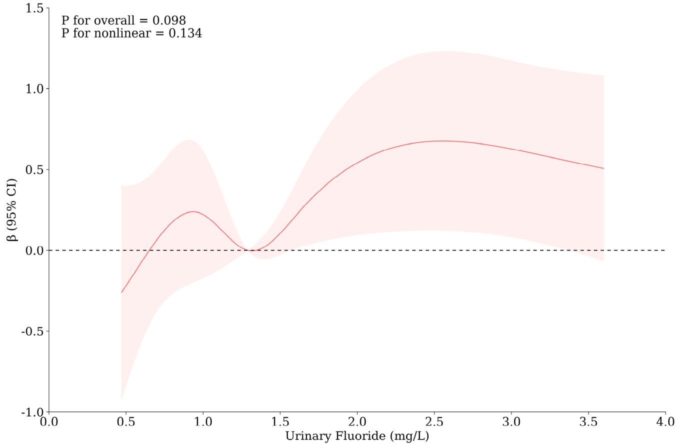

(**C**)

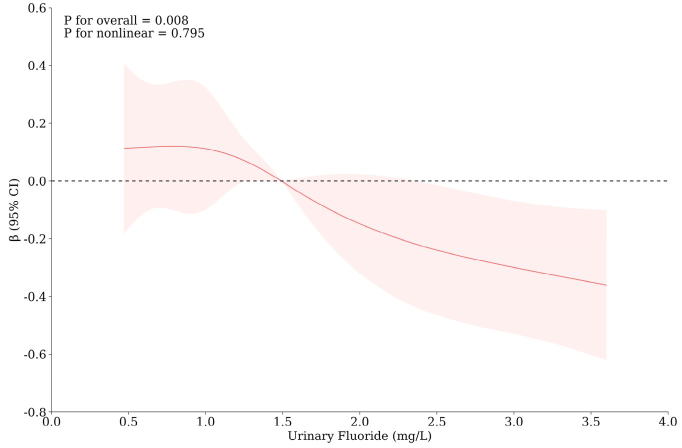

(**D**)

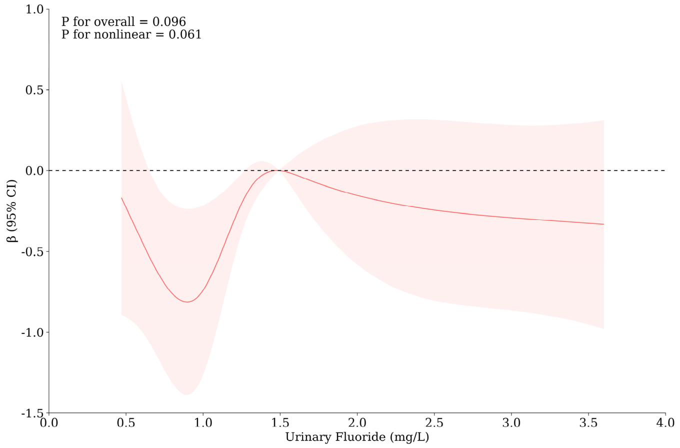
P for overall = 0.096
P for nonlinear = 0.061
β (95% CI)
Urinary Fluoride (mg/L)

(E)

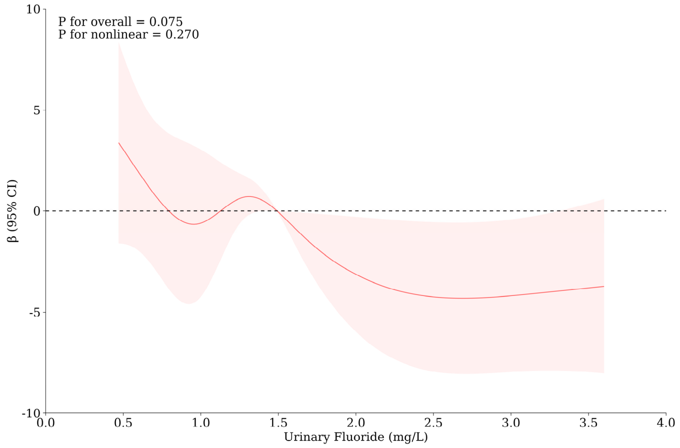
P for overall = 0.075
P for nonlinear = 0.270
β (95% CI)
Urinary Fluoride (mg/L)

(F)

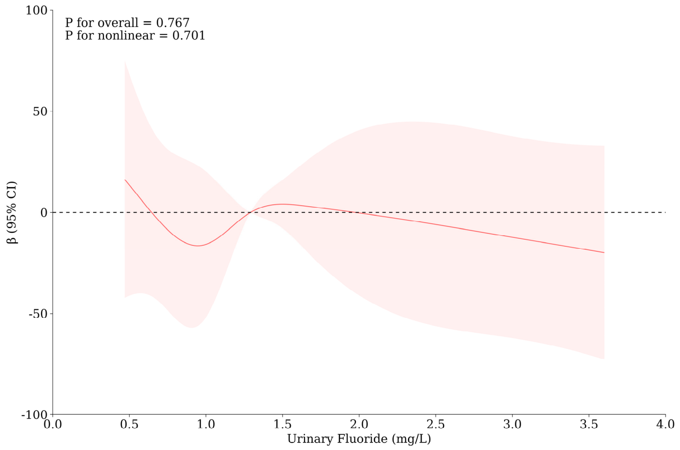
P for overall = 0.767
P for nonlinear = 0.701
β (95% CI)
Urinary Fluoride (mg/L)

(G)

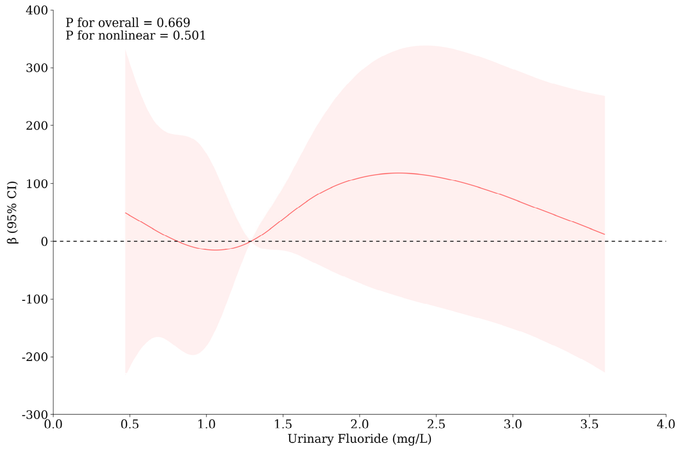
P for overall = 0.669
P for nonlinear = 0.501
β (95% CI)
Urinary Fluoride (mg/L)

(H)

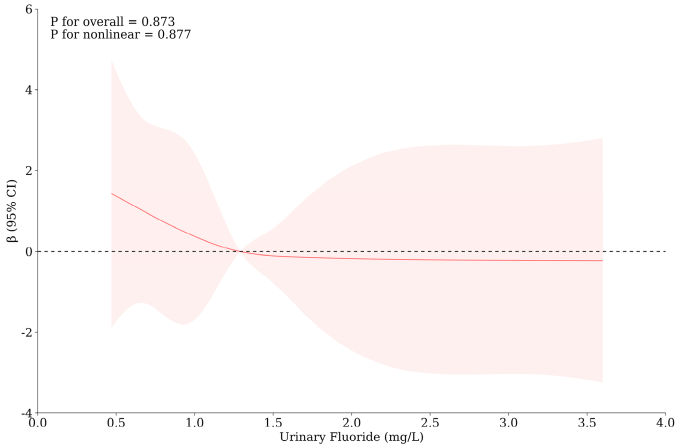

(I)

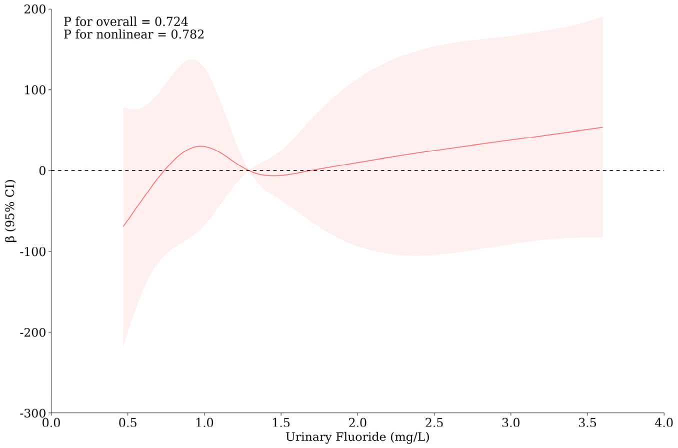

(J)

A. Tvol; B. Thyroid nodule; C. TSH; D. FT3; E. FT4; F. IQ; G. 5-HT; H. NE; I. DA; J. E.

Figure s5. Urinary fluoride RCS curve adjustment model.

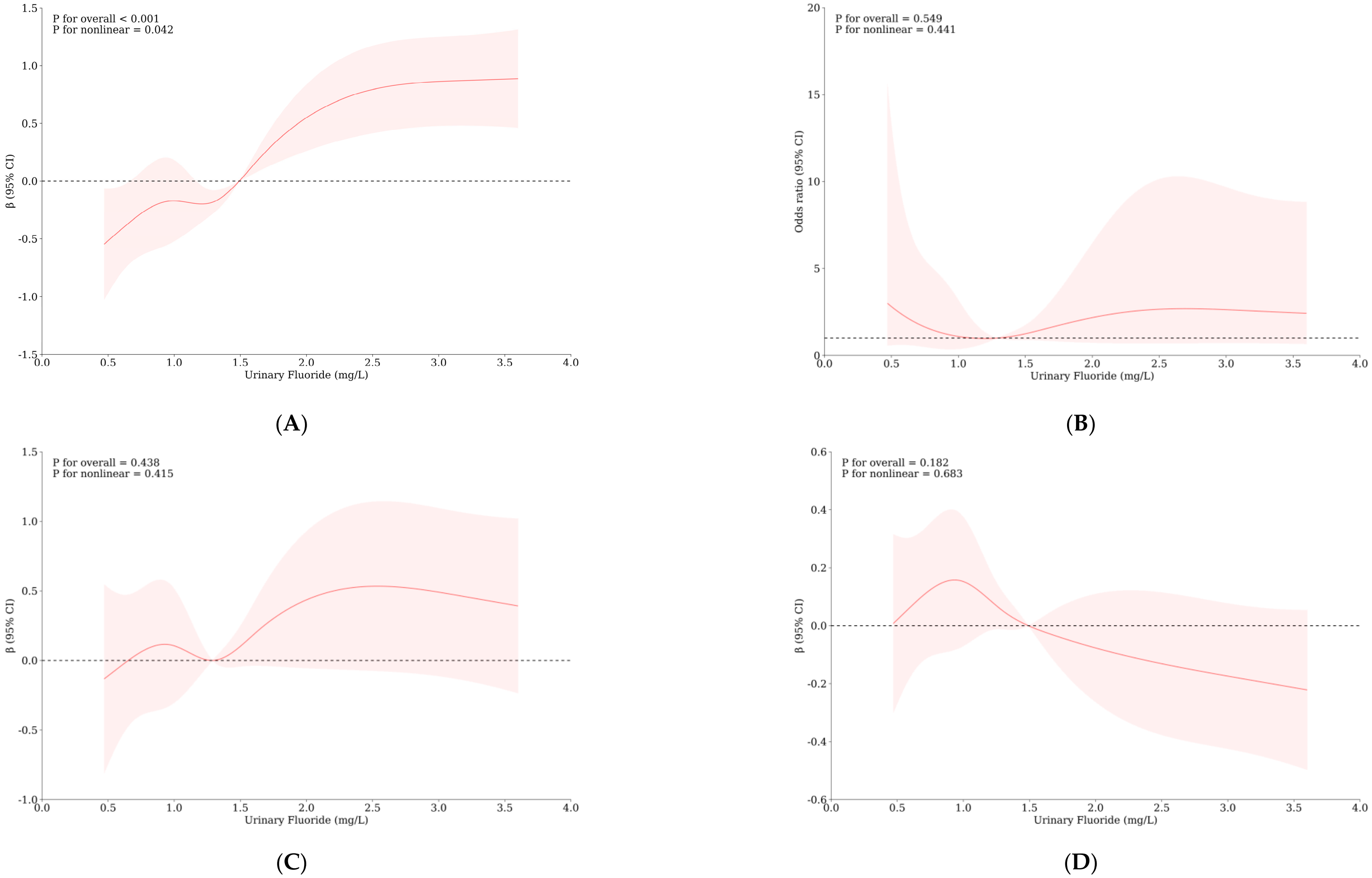

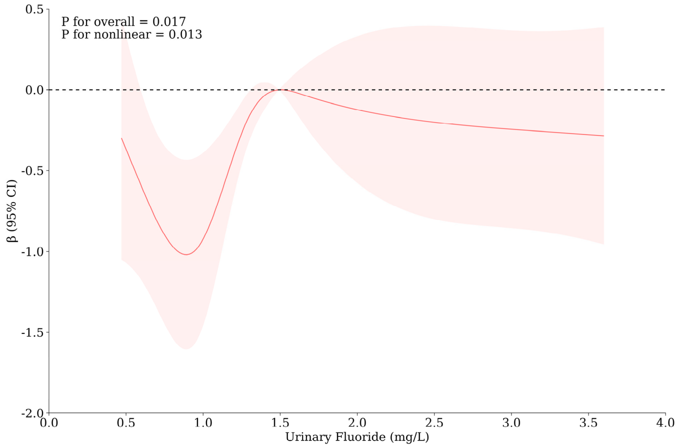
P for overall = 0.017
P for nonlinear = 0.013
β (95% CI)
0.5
0.0
-0.5
-1.0
-1.5
-2.0
0.0
0.5
1.0
1.5
2.0
2.5
3.0
3.5
4.0
Urinary Fluoride (mg/L)

(E)

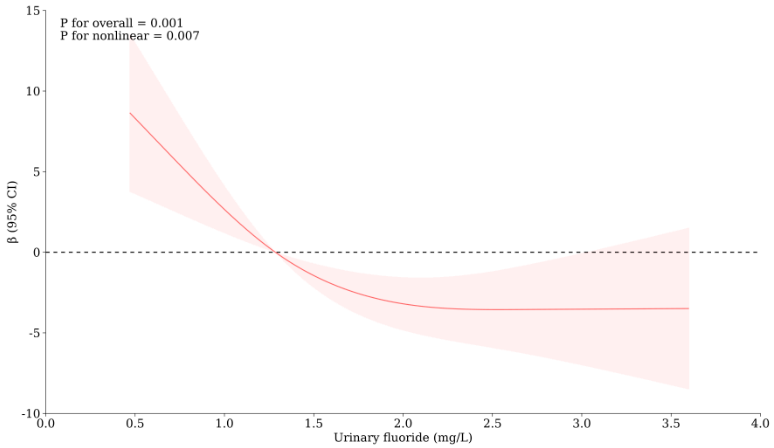
P for overall = 0.001
P for nonlinear = 0.007
β (95% CI)
15
10
5
0
-5
-10
0.0
0.5
1.0
1.5
2.0
2.5
3.0
3.5
4.0
Urinary fluoride (mg/L)

(F)

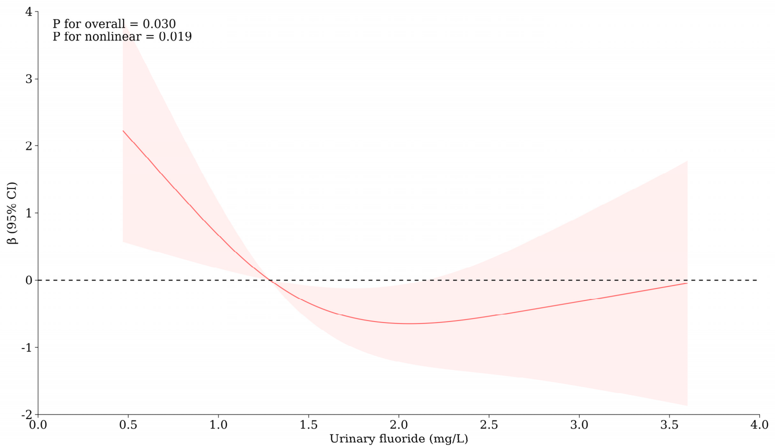
P for overall = 0.030
P for nonlinear = 0.019
β (95% CI)
4
3
2
1
0
-1
-2
0.0
0.5
1.0
1.5
2.0
2.5
3.0
3.5
4.0
Urinary fluoride (mg/L)

(G)

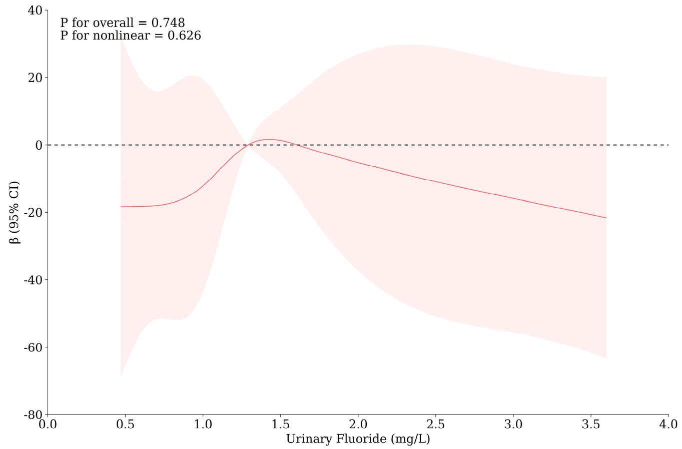
P for overall = 0.748
P for nonlinear = 0.626
β (95% CI)
40
20
0
-20
-40
-60
-80
0.0
0.5
1.0
1.5
2.0
2.5
3.0
3.5
4.0
Urinary Fluoride (mg/L)

(H)

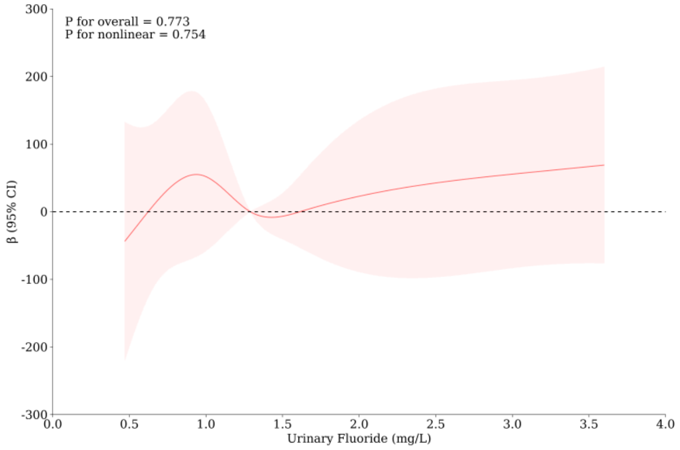

(I)

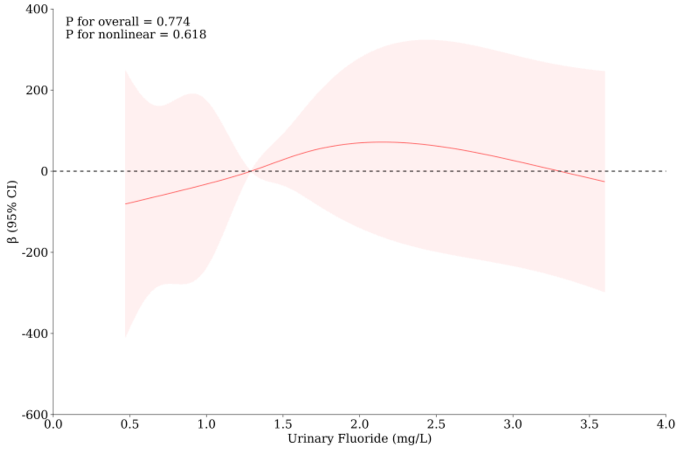

(J)

A. Tvol; B. Thyroid nodule; C. TSH; D. FT3; E. FT4; F. IQ; G. DA; H. 5-HT; I. E; J. NE.
